# Emphysematous Cystitis: An Exuberant Case

**DOI:** 10.7759/cureus.100182

**Published:** 2025-12-27

**Authors:** Sofia Sequeira, Mariana Gradim, Ana Lisa Lima

**Affiliations:** 1 Internal Medicine, Hospital do Santo Espírito Santo da Ilha Terceira, Ilha Terceira, PRT; 2 Immunohemotherapy, Instituto Português de Oncologia do Porto Francisco Gentil, Porto, PRT; 3 Internal Medicine, Hospital Pedro Hispano, Matosinhos, PRT

**Keywords:** computed tomography, emphysematous cystitis, immunosuppression, urinary tract infection, urosepsis

## Abstract

Emphysematous cystitis is a rare but underrecognized urinary tract infection characterized by gas formation within the bladder wall and lumen. It predominantly affects patients with predisposing factors such as immunosuppression and underlying urinary tract abnormalities. Delayed diagnosis may result in severe complications, including bladder necrosis, perforation, and septic shock.

We describe the case of an adult female with an undifferentiated autoimmune disease under long-term corticosteroid therapy and known urinary tract dysfunction, who presented with prostration, abdominal pain, vomiting, and decreased urine output. She rapidly deteriorated into septic shock with multiorgan dysfunction involving the cardiovascular, renal, neurological, and hematological systems. Laboratory findings revealed markedly elevated inflammatory markers and severe acute kidney injury. Computed tomography (CT) of the thorax, abdomen, and pelvis demonstrated emphysematous cystitis with extensive gas tracking into the retropubic and extraperitoneal spaces. A contrast-enhanced study excluded bladder rupture. Broad-spectrum antimicrobial therapy and intensive supportive care were initiated and later tailored based on microbiological cultures isolating *Klebsiella pneumoniae* and *Enterococcus faecalis*. The patient showed progressive clinical, laboratory, and radiological improvement with prolonged conservative management, avoiding surgical intervention.

This case illustrates an unusually extensive radiological presentation of emphysematous cystitis in an immunosuppressed patient with structural urinary tract abnormalities. CT played a pivotal role in diagnosis, exclusion of bladder rupture, and guidance of therapeutic strategy. Despite a severe initial presentation, early multidisciplinary management enabled a favorable outcome without surgical intervention.

Emphysematous cystitis should be considered in septic patients with urinary symptoms and relevant risk factors. Prompt imaging, early targeted antimicrobial therapy, and close multidisciplinary follow-up are crucial to reduce morbidity and mortality, even in severe presentations.

## Introduction

Emphysematous cystitis is a rare and potentially life-threatening form of complicated urinary tract infection characterized by the presence of gas within the bladder wall and lumen [1]. It accounts for approximately 1-2% of all cases of emphysematous infections of the urinary tract and is most commonly reported in elderly patients, particularly those with diabetes mellitus [2,3]. Other well-recognized predisposing factors include immunosuppression, chronic corticosteroid use, neurogenic bladder, urinary tract obstruction, and structural or functional urological abnormalities [4,5].

The pathogenesis of emphysematous cystitis is thought to involve gas-forming microorganisms, most frequently *Escherichia coli* and *Klebsiella pneumoniae*, fermenting glucose or other substrates in a hypoxic environment within the bladder wall [3,6]. Clinical presentation is highly variable, ranging from mild lower urinary tract symptoms to severe sepsis and septic shock, which may delay diagnosis and worsen outcomes [7]. Computed tomography (CT) is considered the imaging modality of choice, allowing accurate detection of intramural gas, assessment of disease extent, and exclusion of complications such as bladder rupture or emphysematous involvement of adjacent tissues [1,8].

Management typically consists of prompt broad-spectrum antimicrobial therapy, bladder drainage, and treatment of underlying predisposing conditions, with surgical intervention reserved for cases complicated by bladder necrosis, perforation, or failure of conservative therapy [2,4]. Despite advances in imaging and antimicrobial therapy, emphysematous cystitis remains associated with significant morbidity and mortality, particularly in patients presenting with sepsis or multiorgan dysfunction [3,7].

We present a case of emphysematous cystitis with unusually extensive extraperitoneal gas extension in an immunosuppressed patient with chronic lower urinary tract dysfunction characterized by impaired bladder emptying and recurrent urinary tract infections, recognized risk factors for emphysematous cystitis, complicated by septic shock and multiorgan failure. This case highlights the diagnostic challenges, the critical role of early imaging, and the potential for successful conservative management even in severe presentations.

## Case presentation

A 32-year-old woman with a history of an undifferentiated autoimmune disease receiving long-term systemic corticosteroid therapy presented to the emergency department with several days of progressive prostration, abdominal pain, nausea, vomiting, and subjective reduction in urine output.

Her past medical history was significant for chronic lower urinary tract dysfunction characterized by impaired bladder emptying with intermittent episodes of urinary retention, managed with intermittent catheterization, without the use of a permanent indwelling urinary catheter. She had a history of recurrent urinary tract infections, previously caused by *Escherichia coli* and *Klebsiella pneumoniae*, with no documented history of extended-spectrum β-lactamase (ESBL)-producing organisms and no recent exposure to antibiotic therapy prior to the current admission.

On arrival, the patient appeared acutely ill and rapidly deteriorated, developing septic shock with multiorgan dysfunction, characterized by profound hemodynamic instability refractory to fluid resuscitation, hypothermia, tachycardia, tachypnea, and altered mental status, requiring immediate resuscitative measures and admission to a critical care setting.

Arterial blood gas analysis demonstrated severe metabolic acidosis with hyperlactatemia, consistent with global tissue hypoperfusion. Laboratory evaluation revealed marked systemic inflammation and rapidly progressive acute kidney injury with oliguria, accompanied by significant electrolyte disturbances. Urinalysis demonstrated marked pyuria, supporting a urinary source of infection. Hematologic dysfunction was evidenced by thrombocytopenia and laboratory features consistent with sepsis-associated coagulopathy.

**Figure 1:**
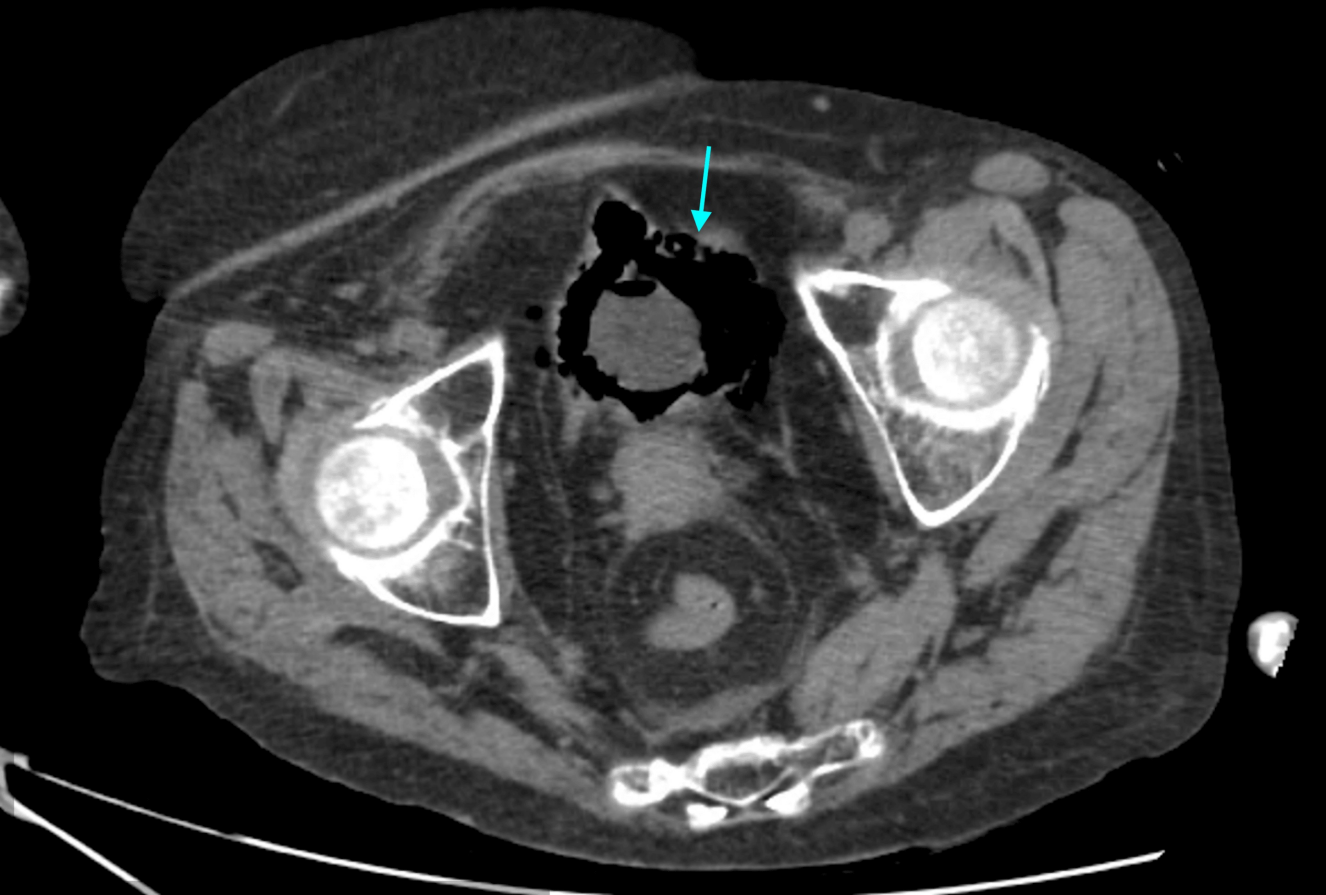
Axial computed tomography image showing circumferential intramural gas within the bladder wall and surrounding perivesical emphysema, characteristic of emphysematous cystitis

**Figure 2:**
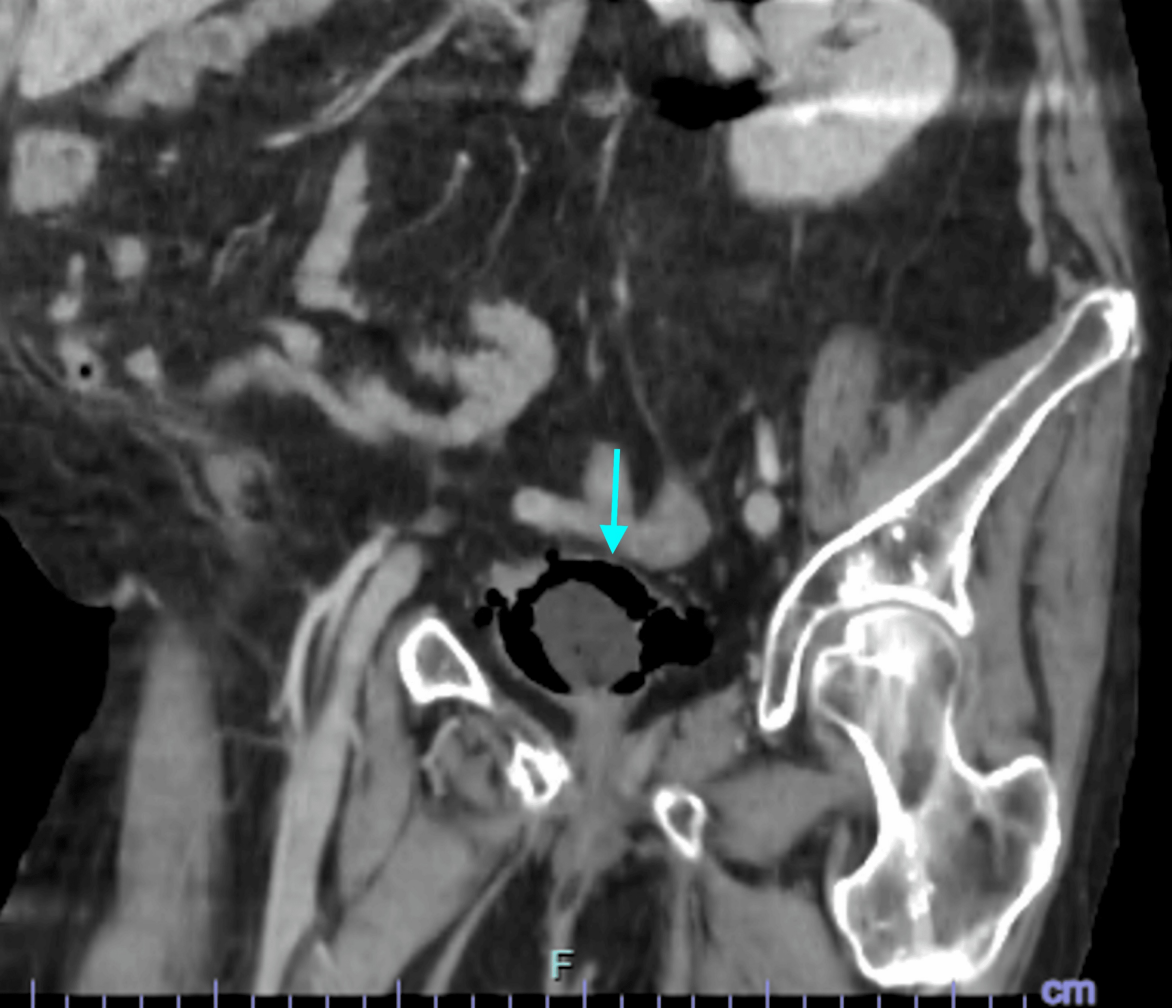
Coronal computed tomography reconstruction demonstrating craniocaudal extension of gas into the retropubic and extraperitoneal pelvic spaces adjacent to the urinary bladder

**Figure 3:**
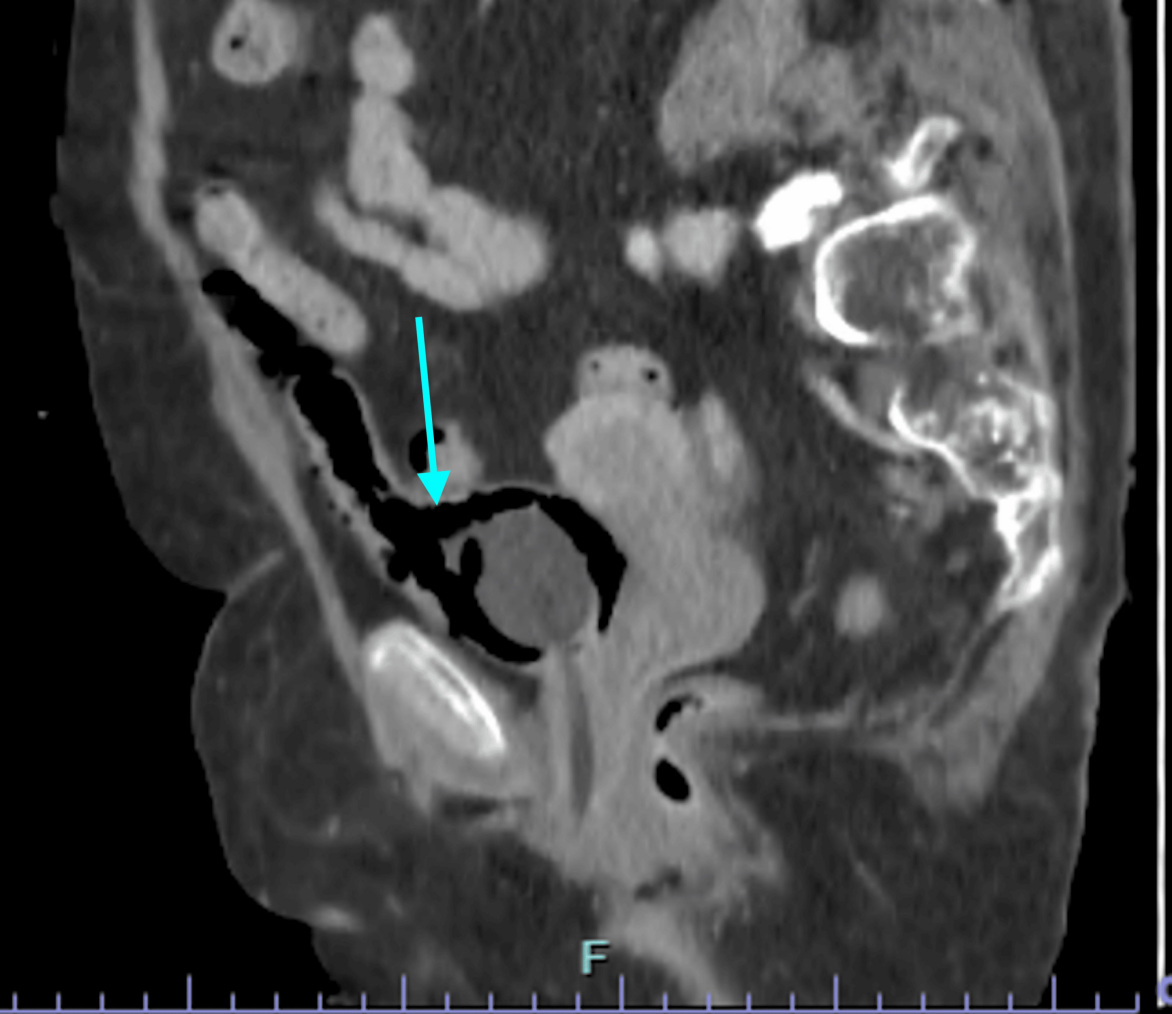
Sagittal computed tomography reconstruction illustrating anterior and posterior extraperitoneal gas collections closely related to the bladder wall, with preservation of the intraperitoneal compartment

In view of the severity of the clinical presentation and concern for a complicated urinary tract infection, urgent cross-sectional imaging was performed. CT of the thorax, abdomen, and pelvis revealed extensive gas within the bladder wall and lumen, consistent with emphysematous cystitis. In addition, marked gas extension was observed in the perivesical tissues, retropubic space, and adjacent extraperitoneal compartments. Axial CT images demonstrated circumferential intramural gas outlining the bladder wall with surrounding perivesical emphysema (Figure 1). Coronal reconstructions illustrated the craniocaudal spread of gas along extraperitoneal pelvic planes (Figure 2), while sagittal images further delineated anterior and cranial extraperitoneal gas collections in close proximity to the bladder dome, without evidence of intraperitoneal involvement (Figure 3). No radiological features suggestive of urachal-related pathology were identified, including the absence of a midline tubular or cystic structure extending from the bladder dome to the umbilicus, and no contrast or urine was observed within a urachal tract on excretory phase imaging.

Based on these findings, a focused differential diagnosis was considered. Emphysematous cystitis was favored due to the presence of circumferential intramural gas outlining the bladder wall with associated intraluminal gas and perivesical emphysema in the context of urinary sepsis. Bladder rupture was excluded by contrast-enhanced CT cystography, which demonstrated intact bladder contours with no contrast extravasation. No CT findings suggested bowel perforation or an enterovesical fistula, as there was no free intraperitoneal air, bowel wall inflammatory changes, fistulous tract, or intravesical fecaloid material. There were also no imaging features of necrotizing infection of the perineum or pelvic soft tissues, no history of recent urinary tract instrumentation or trauma, and no evidence of emphysematous infection involving the upper urinary tract.

Blood and urine cultures were obtained prior to antimicrobial initiation. In the setting of septic shock of presumed urinary origin, empiric broad-spectrum antimicrobial therapy was promptly started, targeting Gram-negative gas-forming organisms and potential *Enterococcus* species. This was combined with aggressive supportive care, including intravenous fluid resuscitation and early vasopressor support with noradrenaline to maintain adequate mean arterial pressure, as well as close hemodynamic and metabolic monitoring in the intensive care unit.

Following microbiological identification of *Klebsiella pneumoniae* and *Enterococcus faecalis*, antimicrobial therapy was subsequently de-escalated and tailored according to susceptibility testing. Adequate bladder drainage and strict urine output monitoring were maintained throughout the acute phase. Mechanical ventilation and renal replacement therapy were not required.

Under multidisciplinary conservative management in the intensive care unit, the patient demonstrated gradual and sustained clinical improvement. Hemodynamic stability was achieved, neurological status normalized, and renal function progressively recovered. Serial imaging studies showed marked regression of the extraperitoneal gas collections and resolution of emphysematous changes. The patient ultimately recovered without the need for surgical intervention and was discharged with appropriate outpatient follow-up.

## Discussion

Emphysematous cystitis is an uncommon but potentially severe form of complicated urinary tract infection, characterized by the accumulation of gas within the bladder wall and lumen [1]. Although most cases are reported in elderly patients with diabetes mellitus, several other predisposing factors have been identified, including immunosuppression, chronic corticosteroid therapy, neurogenic bladder, and structural or functional urinary tract abnormalities, all of which increase susceptibility to infection by gas-forming organisms [2,3]. The present case is noteworthy due to the young age of the patient and the coexistence of multiple nondiabetic risk factors, namely, long-term immunosuppression and chronic urinary tract dysfunction.

The pathophysiology of emphysematous cystitis is thought to involve fermentation of glucose or other substrates by gas-producing bacteria in a hypoxic environment within the bladder wall [3,6]. *Escherichia coli* and *Klebsiella pneumoniae *are the most frequently isolated pathogens, while *Enterococcus *species are less commonly reported but may occur, particularly in immunocompromised hosts [1,6]. In this case, the isolation of *Klebsiella pneumoniae *together with *Enterococcus faecalis* underscores the polymicrobial nature that can be observed in severe presentations and highlights the importance of broad initial antimicrobial coverage.

Clinical presentation is often nonspecific and may range from mild lower urinary tract symptoms to fulminant sepsis and septic shock, contributing to diagnostic delay and increased morbidity [7]. In severe cases, patients may present with multiorgan dysfunction, as observed in our patient, further emphasizing the need for a high index of suspicion in at-risk populations [2,7].

CT is the imaging modality of choice for diagnosis, as it allows accurate detection of intramural and extramural gas, assessment of disease extent, and identification of complications such as bladder rupture or extension into adjacent compartments [1,8]. In the present case, CT imaging revealed an unusually extensive extraperitoneal gas spread, involving the perivesical and retropubic spaces, a finding that raised concern for bladder perforation and necessitated further evaluation with CT cystography.

Management of emphysematous cystitis is primarily conservative and includes prompt administration of broad-spectrum antibiotics, adequate bladder drainage, and correction of underlying predisposing factors [2,4]. Surgical intervention is generally reserved for cases complicated by bladder necrosis, perforation, or failure of medical therapy [4,5]. Despite the severity of presentation and extensive radiological findings, conservative management was successful in this patient, with complete clinical and radiological resolution, supporting previous reports that early diagnosis and aggressive medical treatment can obviate the need for surgery even in advanced cases [4,7].

Although overall mortality associated with emphysematous cystitis is lower than that reported for emphysematous pyelonephritis, outcomes remain significantly worse in patients presenting with sepsis or multiorgan failure [3,7]. This case highlights the importance of early recognition, timely imaging, and multidisciplinary management, particularly in immunosuppressed patients with urinary tract abnormalities, in order to achieve favorable outcomes.

## Conclusions

This case highlights emphysematous cystitis as a potentially life-threatening condition that may present with severe sepsis and multiorgan dysfunction, particularly in patients with immunosuppression and underlying urinary tract abnormalities. It underscores the importance of maintaining a high index of suspicion and the critical role of early CT in establishing the diagnosis, assessing disease extent, and guiding management decisions. Despite an extensive radiological presentation and severe initial clinical course, prompt multidisciplinary care and conservative treatment led to a favorable outcome without the need for surgical intervention. Early recognition and timely management remain essential to improving outcomes in severe presentations of this rare condition.
